# Retraction: 17β-estradiol binding to ERα promotes the progression of prolactinoma through estrogen-response element-induced CaBP-9k upregulation

**DOI:** 10.1042/BSR-2019-1330_RET

**Published:** 2022-12-09

**Keywords:** 17β-estradiol E2, CaBP-9k, ERα, ERE, prolactinoma

This article is being retracted from* Bioscience Reports* at the request of the authors due to the inclusion of an incorrect image in their article. The Editorial Office was also alerted to various concerns from a reader, detailing similarities between various western blots in the manuscript and those found in other manuscripts. These include the Figure 2C ER-alpha, CaBP-9k and GAPDH bands (with Figure 5A Bax from doi: 10.1002/jcla.23193, Figure 5A GAPDH from doi: 10.1155/2020/4817608, and Figure 6C VAV3 from doi: 10.18632/aging.103762 respectively), the Figure 5F GAPDH bands (with Figure 5C A549/GAPDH from doi: 10.1042/BSR20182433 and Figure 4L GAPDH from doi: 10.18632/aging.102325), the Figure 2D CaBP-9k and ER-beta bands, (with Figure 5C SAT1 from doi: 10.18632/aging.103762 and Figure 5B GAPDH from doi: 10.1155/2020/4817608), and the Figure 5F GAPDH bands (with Figure 5 GAPDH bands from doi: 10.1042/BSR20160521). The authors are unable to correct the article and wish to retract the article. The Editor-in-Chief and Editorial Board agree with the retraction.

